# Emergence and Genomic Characterization of a *spa* Type t4407 ST6-SCC*mec* Type IVa Methicillin-Resistant *Staphylococcus aureus* Strain Isolated from Al-Karak Hospital, Jordan

**DOI:** 10.3390/medicina60020295

**Published:** 2024-02-09

**Authors:** Yasser Gaber, Heba M. TumAllah, Nourhan H. AbdelAllah, Wael A. Al-Zereini, Mohammad A. Abu-Lubad, Amin A. Aqel, Walid F. Elkhatib, Richard V. Goering, Ahmed M. Soliman

**Affiliations:** 1 Department of Pharmaceutics and Pharmaceutical Technology, Faculty of Pharmacy, Mutah University, Al-Karak 61710, Jordan; heba_tumallah@yahoo.com; 2 Department of Microbiology and Immunology, Faculty of Pharmacy, Beni-Suef University, Beni-Suef 62511, Egypt; nourhan.hassan@pharm.bsu.edu.eg; 3 Clinical Trials Department, Central Administrative of Biological, Innovative Products and Clinical Trials, Egyptian Drug Authority, Giza 12654, Egypt; 4 Department of Biological Sciences, Faculty of Sciences, Mutah University, Al-Karak 61710, Jordan; wzereini@mutah.edu.jo; 5 Department of Microbiology and Pathology, Faculty of Medicine, Mutah University, Al-Karak 61710, Jordan; abu_lubbad@mutah.edu.jo (M.A.A.-L.); aminaq@mutah.edu.jo (A.A.A.); 6 Microbiology and Immunology Department, Faculty of Pharmacy, Ain Shams University, African Union Organization St., Abbassia, Cairo 11566, Egypt; walid-elkhatib@gu.edu.eg; 7 Department of Microbiology & Immunology, Faculty of Pharmacy, Galala University, New Galala City, Suez 43713, Egypt; 8 Department of Medical Microbiology and Immunology, Creighton University School of Medicine, Omaha, NE 68178, USA; richardgoering@creighton.edu; 9 Department of Microbiology and Immunology, Faculty of Pharmacy, Kafr-Elsheikh University, Kafr El-Sheikh 33516, Egypt; ahmed_soliman@pharm.kfs.edu.eg

**Keywords:** Jordan, Middle East, whole-genome sequencing, MRSA, *spa*, SCC*mec*, ST6, hospital

## Abstract

*Background and Objectives*: Methicillin-resistant *Staphylococcus aureus* (MRSA) is a major concern in Jordanian hospitals in terms of infection control. The purpose of this study was to identify the resistance patterns of *Staphylococcus aureus* strains isolated from surfaces of critical locations within the Al-Karak Governmental Hospital in 2019. Additionally, the study aimed to conduct whole-genome sequencing on the isolates. *Materials and Methods*: In February 2019, fourteen *S. aureus* strains were isolated from surfaces in critical sites in the Al-Karak Governmental Hospital. These isolates underwent antibiogram testing to determine their resistance profile. Genome sequencing using the Illumina MiSeq platform was applied to the extracted DNA from these isolates. The genomic data, including coding sequences, were analyzed to identify lineage, resistance genes, and plasmids. *Results*: The antibiogram results revealed that 11 of the 14 isolates were resistant to oxacillin, 6 to linezolid, and 1 to rifampicin, while none showed resistance to chloramphenicol. Eleven isolates were identified as MRSA, with a novel spa type (t4407) not previously reported in Jordan. High-quality sequencing data were obtained for only one isolate, i.e., A29, the genome showed 2,789,641 bp with a 32.7% GC content and contained 2650 coding sequences. Genomic analysis indicated the ST6 lineage, *mecA* gene (SCC*mec* type IVa(2B)), and a hybrid plasmid (pJOR_*blaZ*) carrying the *blaZ* gene for β-lactam resistance. Genomic data were deposited in NCBI (CP104989). The A29 genome closely resembled an MRSA genome isolated from a Danish hospital in 2011. The SNP analysis revealed identical antimicrobial resistance genes in these two genomes. *Conclusions*: This study unveils the first genomic sequence of an MRSA isolate from Jordan, marked by distinctive genotypic traits. The findings enhance our understanding of the MRSA types circulating in Jordan and the region and substantiate the phenomenon of intercontinental MRSA transmission.

## 1. Introduction

*Staphylococcus aureus,* a Gram-positive bacterium that primarily infects humans and animals, can cause minor skin diseases such as scalded skin, impetigo, boils, pimples, and abscesses [1]. It also causes fatal diseases such as endocarditis, bacteremia, meningitis, pneumonia, and sepsis [1]. Methicillin-resistant *S. aureus* (MRSA) is a growing global health threat and was recognized by the WHO as a priority 2 ‘high’ pathogen on its first list of priority pathogens [2]. In the United States, in 2017, MRSA was estimated to cause 323,700 infections in hospitalized patients with 10,600 estimated deaths and USD 1.7 billion as an estimate of the healthcare cost [3]. MRSA is also among ESKAPE pathogens that can spread antimicrobial resistance genes through horizontal gene transfer (HGT) via mobile genetic elements (MGEs) [4]. One of the most important examples of MGEs in MRSA is the *Staphylococcal* cassette chromosome (SCC*mec*) carrying the *mec* gene (*mecA*, *mecB*, and *mecC*) that encodes a specific penicillin-binding protein (PBP2a) leading to the β-lactam resistance, and site-specific recombinase genes *ccrAB* or/and *ccrC* mediating correct excision and integration of SCC*me* [5]. 

Despite enhanced preventive measures and surveillance, MRSA remains an enduring challenge in Jordanian healthcare settings. Limited published reports exist to unveil the genotypes of MRSA in Jordan, indicating a significant gap in our understanding of the specific genetic characteristics of circulating strains [6,7,8,9,10,11,12,13,14,15,16,17]. Alzoubi et al. (2014) investigated 210 nasal swabs from children aged 6–11 years and predominantly identified SCC*mec* type IV, with t223 as the primary *spa* type [18]. Bazzoun et al. (2014) studied 60 hospital isolates and found t044 to be the most common *spa* type of isolated MRSA strains, although the specific type of SCC*mec* was not determined [19]. Another study by Aqel et al. examined 716 nasal swabs from healthcare workers, adults, and children, primarily discerning SCC*mec* types IVa and Vc and highlighting t223 as the dominant MRSA *spa* type [13]. Focusing on the health implications in diabetic patients, Al-Bakri et al. examined 87 diabetic foot ulcer samples, identifying SCC*mec* type IVe as the predominant type of MRSA, and the percentage distribution of *spa* types among isolates as t9519 (76%), t223 (14·7%), and t044 (5·9%) [9]. Khalil et al. analyzed *S. aureus* isolates from children in Jordan using genotyping techniques such as MLST. Their findings highlighted the predominance of the ST80 type in MRSA strains. Specifically, ST80-SCC*mec* type IV emerged as the dominant strain [20]. 

The use of SCC*mec* and *spa* typing has been in use for the last two decades for MRSA classification and epidemiological studies [5,21,22]. However, advancements in whole-genome sequencing (WGS) technology and the continuous reduction in its cost have made it an attractive alternative to conventional typing methods. WGS not only enhances precision but also provides a comprehensive understanding of genetic relatedness in MRSA and other bacteria. While admitting the importance of WGS, PCR-based methods will still play a role in molecular epidemiology due to their simplicity and cost effectiveness. Adhering to nomenclature rules for SCC*mec*, especially for diverse isolates, ensures a seamless transition to advanced technologies. Whole-genome sequencing of MRSA isolates will improve diagnosis, management, and infection reduction of MRSA [21,23,24,25,26].

In order to better understand the genetic traits and epidemiology of MRSA strains in Jordan, it is crucial to conduct whole-genome sequencing on a larger scale to characterize MRSA isolates. The aim of the current study was to analyze MRSA strains that were found on critical surfaces within the Al-Karak Governmental Hospital. This involved conducting antibiogram testing on the isolated *S. aureus* strains, followed by whole-genome sequencing and bioinformatic analysis. Our main goal was to identify strain typing, resistance, and virulence genes. The broader objective of this research was to enhance our understanding of the genetic characteristics and resistance mechanisms of MRSA in the region.

## 2. Materials and Methods

### 2.1. Media

Blood agar, mannitol salt agar, and antibiotic susceptibility discs were obtained from Oxoid Ltd., Basingstoke, Hampshire, UK. MacConkey’s Agar was obtained from Conda SA laboratories, Pronadisa, Spain. The brain-heart infusion broth was obtained from Alpha Chemika, Mumbai, India. The Muller Hinton broth and Muller Hinton agar were purchased from Biolab, Budapest, Hungary. Lastly, lysostaphin was sourced from Sigma-Aldrich, Eschenstrasse 5, D-82024 TAUFKIRCHEN, Germany.

### 2.2. Sample Collection

The surveillance of the MRSA cohort was conducted in February 2019 at the Al-Karak Governmental Hospital. Samples were collected from hospital surfaces, including door handles from the doors of the intensive care unit, basin handles in the outpatient clinics, blood bank, and elevator surfaces. The samples were obtained by rubbing and rotating sterile swabs moistened with nutrient broth. Then, they were immediately inoculated on the surface of 5% blood agar, MacConkey agar plates, and Mannitol salt agar (MSA) plates, and incubated at 37 °C for at least 48 h. The preliminary identification of the *Staphylococcal* isolates was based on the yellow colonies obtained on MSA, the positive catalase test, and the Gram staining showing Gram-positive cocci [27]. A total of 14 preliminary identified *S. aureus* isolates were recorded.

### 2.3. Antimicrobial Susceptibility Testing

The antimicrobial susceptibility testing was carried out using the disc diffusion method following the National Committee for Clinical Laboratory Standards [28]. Fresh *S. aureus* isolates were prepared by inoculating a loopful of bacterial suspension in sterile Mueller–Hinton broth and incubating at 37 °C for 2–4 h until the turbidity was consistent with a 0.5 McFarland standard. The bacterial suspension was then evenly spread on Mueller–Hinton agar plates. The antibiotic discs tested included ampicillin (10 μg), cefotaxime (30 μg), cefoxitin (30 μg), oxacillin (1 μg), ceftazidime (30 μg), clindamycin (2 μg), linezolid (30 μg), amikacin (30 μg), gentamicin (10 μg), rifampicin (5 μg), and chloramphenicol (30 μg). The diameter of the inhibition zone of each antimicrobial was measured and classified as susceptible, intermediate, and resistant. *S. aureus* ATCC25923 was used as a control for the experimental conditions [29].

### 2.4. DNA Isolation and Whole-Genome Sequencing (WGS)

Whole-genome sequencing (WGS) was performed for the 14 isolates; however, high-quality data were obtained for only one isolate, i.e., A29. The DNeasy kit obtained from Qiagen (Hilden, Germany) and containing buffers solution labeled as lysis buffer, AL, AW1, AW2, and AE was used to extract the DNA, and all *staphylococcal* isolates were cultured in brain-heart infusion broth at an optical density (OD) of 1.0 to ensure an adequate concentration of bacterial cells for downstream processing. The culture obtained was centrifuged, and the bacterial pellets were collected. Bacterial cells, approximately equivalent to 2 × 10^9^ cells or ~1.0 mL of OD 1.0 culture, were harvested for the cell harvesting and lysis step. These cells were transferred to a microcentrifuge tube and subsequently centrifuged for 10 min at 5000× *g*. The supernatant was discarded following this centrifugation, ensuring that the bacterial pellet was left undisturbed. The obtained pellet was then resuspended in 180 μL of a freshly prepared lysis buffer. This buffer consisted of 2.25 mL of TE buffer (10 mM Tris HCl and 1 mM EDTA), 30 μL Triton X-100, 250 μL Lysostaphin (0.05 μg/mL), and 50 mg of Lysozyme (20 mg/mL final concentration). The obtained mixture was incubated at 37 °C for 1 h, with vortexing performed every 15 min to promote thorough mixing. At the end of the incubation period, 200 μL of AL buffer from the Qiagen DNeasy kit was added to the mixture. The solution was then vortexed for around 15 s. After ensuring a uniform mixture, the tube was placed in a 56 °C water bath for 30 min, with a vortex at the midpoint to guarantee consistent exposure to the heat. Following this step, 200 μL of ethanol (99% purity) was added to the mixture. This mixture was then thoroughly mixed using a vortex. The entire solution, including any precipitates, was transferred to a labeled DNeasy mini-column located within a 2 mL collection tube. Centrifugation was followed for 1 min at 6000× *g*. After centrifugation, the flow-through was discarded, and the DNeasy mini-column was transitioned to a fresh 2 mL collection tube. To the column, 500 μL of buffer AW1 was added, which was followed by 1-min centrifugation at 6000× *g*. This flow-through was also discarded, and the column was transferred to a new collection tube. The exact process was repeated using 500 μL of buffer AW2, but this time centrifuged at 20,000*× g* for 3 min to ensure the membrane was sufficiently dried. In the DNA elution phase, the column was moved to a microcentrifuge tube. Directly to the DNeasy membrane, 200 μL of buffer AE was added and allowed to sit at room temperature for one minute. After incubation, centrifugation at 6000 ×*g* for 1 min was performed to elute DNA. Care was taken while discarding the column to prevent the transfer of any flow-through. Finally, the successfully isolated DNA was securely stored at −20 °C, ready for shipping to the USA for sequencing.

For WGS, we prepared the DNA library using a Nextera XT library preparation kit and a Nextera XT index kit (Illumina, San Diego, CA, USA) following the manufacturer’s instructions. Sequencing was performed on an Illumina MiSeq instrument in compliance with the manufacturer’s protocol. Bioinformatic analysis of raw sequencing reads, including quality control measures to filter out low quality, was performed using BioNumerics v8.1 (BioMerieux, Sint-Martens-Latem, Belgium), genome assembly using the SPAdes algorithm, and subsequent analysis was performed using default BioNumerics parameters. 

### 2.5. Genome Annotation 

The final draft genome sequence was annotated using RAST [30,31] and the NCBI Prokaryotic Genome Annotation Pipeline (PGAP) [32]. The annotated genes from the RAST server were downloaded as a Microsoft Excel sheet, and their genomic characteristics were compared. The RAST server was used to retrieve antibiotic-resistant genes for *S. aureus* A29, after which a comparison was performed. Antimicrobial resistance and virulence genes associated with their genomic location were detected using ResFinder 4.4.2 [33], VirulenceFinder 2 [34,35,36,37], and MobileElementFinder tools [38]; these tools are available from the Centre for Genomic Epidemiology (www.genomicepidemiology.org/services/), accessed on 1 November 2023. By default, the analyzed genome was uploaded with the default settings. The plasmid Inc groups, multilocus sequence typing (MLST), spa typing, and SCC*mec* typing were identified using the PlasmidFinder, MLST 2.0 software, spaTyper [39], and SCCmecFinder-1.2, respectively. These tools are also available at the Centre for Genomic Epidemiology (www.genomicepidemiology.org/services/), accessed on 1 November 2023. To compare the complete sequence of the plasmid carrying the *bla-Z* gene, the BRIG tool was used [40]. 

### 2.6. Single Nucleotide Polymorphism (SNP) Analysis 

The CSIPhylogeny tool, developed by the Centre for Genomic Epidemiology (www.genomicepidemiology.org/services/), accessed on 14 January 2024, at DTU in Lyngby, Denmark, was employed for the identification of single nucleotide polymorphisms (SNPs) within the A29 genome to the CP047021 reference genome [41]. The tool default settings were used: a minimum depth of 10, a relative depth of 10, and a minimum SNP quality of 30. This comparative analysis involved aligning the genomic sequence of the A29 isolate with the established Danish reference genome (CP047021). The resultant SNP calls, formatted in variant call format (VCF), were acquired and subsequently juxtaposed with the cataloged antimicrobial resistance (AMR) genes within the CP047021 genomes. 

## 3. Results

### 3.1. Sources and Antibiograms of the Isolates

**Figure 1:**
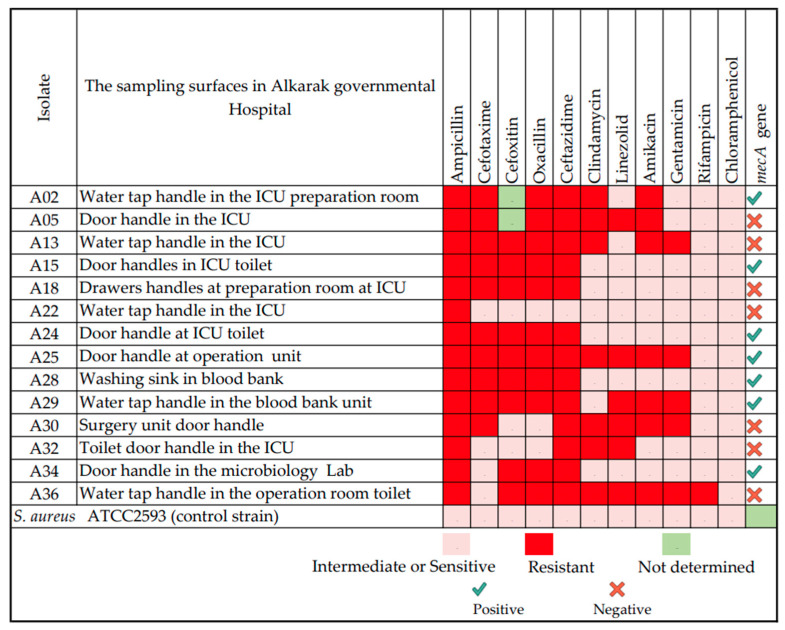
Antibiotic resistance profiles of *S. aureus* isolates sampled from surfaces within the Al-Karak Governmental Hospital, Jordan (February 2019 cohort sampling).

The current study was conducted in a cohort sampling in February 2019 at Al-Karak Hospital, Karak Governorate, Jordan. In the study, we isolated 14 *S. aureus* strains from surfaces of critical areas located within the Al-Karak Governmental Hospital. The study sampled various surfaces including the handles of water taps, doors, and drawers inside the ICU, blood bank unit, and the microbiology lab. The 14 *S. aureus* strains exhibited varying resistance patterns against several antibiotics. The resistance patterns for the 14 isolates were as follows: ampicillin (100%), cefotaxime (71.4%), oxacillin (78.5%), ceftazidime (92.9%), clindamycin (50%), linezolid (42.9%), amikacin (50%), gentamicin (35.7%), and rifampicin (7%). Nine out of twelve isolates showed resistance to cefoxitin (0.69%), while all isolates demonstrated susceptibility to chloramphenicol (Figure 1). The isolate A29 showed multidrug resistance phenotypes resistant to ampicillin, amikacin, cefotaxime, cefoxitin, ceftazidime, gentamicin, linezolid, and methicillin. It showed susceptibility to chloramphenicol, clindamycin, and rifampicin. 

### 3.2. Whole-Genome Sequencing Results and A29 Genome Annotation 

The preliminary evaluation of the WGS data for the 14 isolates of *S. aureus* showed that 9 (50%) were *mec*A positive (Figure 1); also, 13 isolates (92.9%) were shown to be *spa* type t4407. The study aimed to sequence the entire genome of all isolates. However, high-quality sequencing data were only obtained for A29, which was deposited in NCBI under the accession number CP104989 (Table 1). The genomic sequence consisted of 2,789,641 bp, with a GC content of 32.7%. The number of coding and RNA sequences was 2650 and 67, respectively. The quality assessment revealed an N50 value of 53,762 bp, signifying that half of the genome is represented by contigs of this length or longer. The low L50 value of 19 bp indicates the high quality of the obtained genomic data, reflecting the average contig length needed to cover half of the genome. The quality criteria were N50: 53,762 and L50 of 19 bp length. BLAST analysis (blastn) of the A29 genome to nucleotides database at NCBI identified the nearest hit, a strain M2024 (accession number CP047021.1), isolated from a skin infection, (https://www.ncbi.nlm.nih.gov/biosample/SAMN13612188), accessed on 1 January 2024, of a patient in a Danish hospital in 2011 [42]. Genome annotation was performed using the RAST server. It showed 1243 genes divided into 27 subsystems, including cofactors, cell wall, virulence, and phages, as illustrated in Figure S1. The strain carried two antibiotic resistance genes against β-lactams, i.e., *mecA* and *bla*Z; Furthermore, it incorporated 12 virulence genes, i.e., *aur*: aureolysin; *lukD*: leukocidin D component; *lukE*: leukocidin *E* component; *spl*A: serine protease *SplA*; *splB*: serine protease *SplB*; *splE*: serine protease *SplE*; *hlgA*: gamma-hemolysin chain II precursor; *hlgB*: gamma-hemolysin component B precursor; *hlgC*: gamma-hemolysin component C; *sak*: staphylokinase; *scn*: staphylococcal complement inhibitor; *sea*: enterotoxin A (Table S1, Supplementary Information).

### 3.3. Typing Results of A29 and Plasmids 

**Figure 2:**
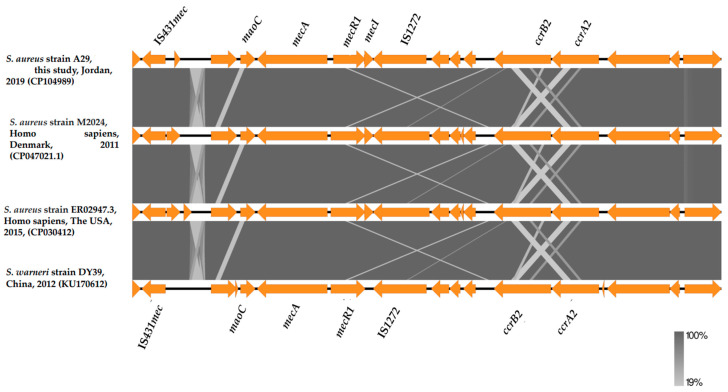
Schematic representation of SCC*mec* type IVa identified in the A29 MRSA strain (this study) along with other closely related sequences (CP047021.1, CP030412, and KU170612) in the GenBank NCBI database. The gray-scale connections indicate the level of sequence homology, with the intensity corresponding to the percentage of similarity.

Multilocus type sequencing (MLST) was performed by uploading the obtained WGS FASTA file to the MLST-2.0 server. The results indicated that the strain belonged to ST6 (*arc*C 12, *aro*E 4, *glp*F 1, *gm*k 4, *pta* 12, *tpi* 1, and *yqi*L 3). The *spa* type of our strain was detected using the spatyper [39]. The strain has *spa* type t4407 (Table 2). The acquisition of mobile SCC*mec* is a defining feature of MRSA that exhibits β-lactam resistance [22]. The strain was positive for SCC*mec* type IVa (2B) (Table 2). The prediction of SCC*mec* in the A29 genome according to homology with the database sequences is shown in Table 2. SCCmecFinder-1.2 identified that this SCC*mec* contained *ccrA2* and *ccrB2* as a *ccr* gene complex and *mecA*, *dmecR1*, and *IS1272* as a *mec* gene complex. The expected whole SCC*mec* element in A29 has a 99.99% identity with 100% coverage to SCC*mec* type IVa identified in (i) an MRSA strain M2024 identified from a patient in Denmark in 2011 (GenBank: CP047021.1), (ii) the MRSA strain ER02947.3 identified from human blood sample in the USA in 2015 (GenBank: CP030412), and (iii) the *S. warneri* strain DY39 detected in China from milk of dairy cow with mastitis in 2012 (GenBank: KU170612) (Figure 2).

### 3.4. Identification of Plasmids in the A29 Genome 

**Figure 3:**
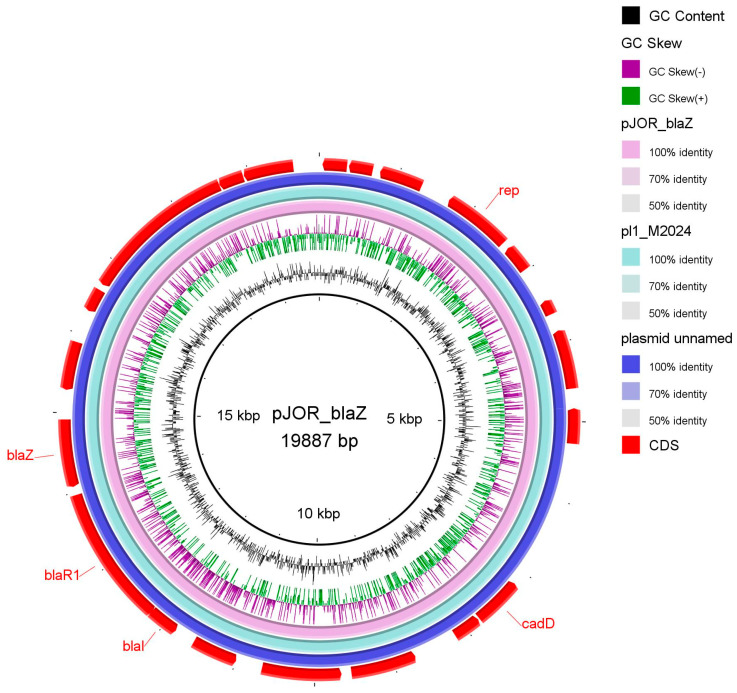
Schematic representation of pJOR_*blaZ* plasmid identified from the MRSA strain A29 compared to the two nearest hits in the NCBI nucleotide database: pl1_M2024 (CP047022.1) and plasmid unnamed (CP062377.1). Red lines denote coding sequence (CDS), the figure was created with the BRIG tool.

The PlasmidFinder 2.1 tool identified one hybrid plasmid (rep5a and rep16) named pJOR_*blaZ*. The blastn results of the plasmid to the nucleotide NCBI database showed almost identical hits as follows: (i) plasmid pl1_M2024 identified in the human clinical strain M2024 in Denmark in 2011 (CP047022.1); (ii) unnamed plasmid in the human clinical strain NAS_AN_099 in the USA (CP062377.1). The alignment of the pJOR_*blaZ* to these hits is shown in Figure 3. The plasmid replicons of isolate A29 obtained from PlasmidFinder 2.1 demonstrated two different replicons, rep5a and rep16, belonging to the replicon families Rep3 and Inc18, respectively (Table 2). The *blaZ* gene is located in position 2694466 to 2695311 of the A29 genome (Table 2). 

### 3.5. SNP Analysis 

The detected single nucleotide polymorphisms (SNPs) in the A29 genome were systematically aligned with the positions of antimicrobial resistance (AMR) genes in the Danish reference genome (CP047021). Intriguingly, all identified SNPs were located outside the annotated AMR genes, as shown in Table S2 (Supplementary Information). A notable exception (position 2771818 in CP047021, SNP A to G) was observed, where an SNP occurred within the collagen adhesin *cna* gene, recognized as a virulence factor (Table S2).

### 3.6. Comparison of Current Findings to Previous Reports 

Table 3 presents a comparison between the genotypes of MRSA that were reported in Jordan over the past decade and the results of the current study. There have been very few studies on the genotypes present in the Jordanian environment. A literature search showed reports of only two MLST lineages (ST80 and ST997) in a study published in 2014 [20,43]. Interestingly, the comparison revealed a new spa type, t4407, which was not previously recorded in published studies. Table 3 displays the spa types reported in Jordan over the past decade [9,13,15,16,43]. 

## 4. Discussion 

In February 2019, a sampling campaign was conducted to investigate the presence of MRSA on the surfaces of the critical sites inside Al-Karak Hospital, Al-Karak Governorate, Jordan. Among the 14 isolates identified, 11 were confirmed as MRSA through antibiograms. Whole-genome sequencing was performed on all 14 isolates, which revealed a novel spa type t4407. However, only one isolate (A29) produced high-quality sequencing results. This A29 isolate was collected from the surface of the tap water handle in the blood bank unit. A29 was found to be multidrug resistant and was resistant to all antimicrobials tested except chloramphenicol, rifampicin, and clindamycin.

The MLST of A29 showed that the profiles of the seven detected alleles belong to ST6, and its *spa* type was t4407, which is very similar to the t304/ST6 MRSA isolates reported in northern Europe (e.g., Denmark, Norway, and Iceland) [42]. This *spa* type t304/ST6 was identified in the DK2011-01 isolate (accession number: CP047021). The *spa* type t4407 is related to t304 but missing repeats 34 and 24 in the middle of the sequence. The A29 and CP047021 comparison exhibited 99% query coverage and 99.96% identity. The SNP analysis indicates a strong correlation between the A29 genome and CP047021 genome, with the conservation of AMR genes. This provides evidence of intercontinental spread of MRSA strains. Indeed, Bartels et al. illustrated the common description of this MRSA clone (*spa* type t304/ST6) and indicated its European emergence because of the influx of Syrian refugees to Europe from the civil war [42]. 

The *Staphylococcal* cassette chromosome (SCC*mec*), a mobile element that carries the main genetic factor for broad-spectrum β-lactam resistance, is a characteristic feature of MRSA. SCC*mec* typing is a helpful method for assessing MRSA strain relatedness and issues related to the genomic basis of methicillin resistance. *Staphylococcal* lineages resistant to methicillin have emerged due to the acquisition and insertion of the SCC*mec* element on the chromosomes of some susceptible strains [22]. SCC*mec* contained mainly (i) the *mec* gene (*mecA*, *mecB*, and *mecC*) that encodes a specific penicillin-binding protein (PBP2a) and (ii) site-specific recombinase genes (*ccrAB* and/or *ccrC*) mediating excision and integration. It is widely recognized that SCC*mec* types IV and V are familiar types of community-acquired MRSA. In contrast, SCC*mec* types I, II, and III are commonly associated with hospital-acquired MRSA [24]. A29 harbored SCC*mec* type IVa (containing *ccrA2*/*ccrB2* and *mecA*); this type was often detected in infection with community-associated MRSA (CA-MRSA). SCC*mec* type IVa MRSA was the most prevalent strain in a study performed by Khalil et al.; samples were collected from Jordan University Hospital in Amman, Jordan, in the period between August and October 2008 [20]. Previous reports of MRSA isolated in Jordan showed that the prevalent SCC*mec* was type IVe, specifically among diabetic foot ulcer infections [9]. Another study reported the prevalence of type IV and V among MRSA strains [13]. Tabaja et al. have shown that CC6 was detected in some infective MRSA isolates from the Kingdom of Saudi Arabia (KSA) and Kuwait. In KSA, CC6 represented 13% of Riyadh-infectious isolates and was associated with SCC*mec* type IV, typically found in community-acquired MRSA. In Kuwait, CC6 represented 4% of Kuwait City infective isolates and was associated with SCC*mec* type IV [45].

A few studies have reported MRSA genotypes circulating in Jordan (Table 3). In a major hospital in Amman, *spa* type t044 was identified as the most common among MRSA strains [19]. Furthermore, Harastani et al. showed in the heterogenicity among the type IV MRSA clonal complex (CC80-MRSA-IV) in Jordan and Lebanon and noted the dominance of the *spa* type t044 [43]. More recently, attention has been shifted to MRSA in livestock, particularly cattle suffering from subclinical mastitis. A new spa type (t17158) was identified as a significant cause of mastitis in Jordanian dairy cows, signaling a potential new reservoir for MRSA [16]. These findings show the different settings in which MRSA is found in Jordan and emphasize the importance of continuous surveillance to understand the changing epidemiology of MRSA, which is crucial for effective infection prevention and control. 

Our data showed the presence of *mecA* on the chromosome and *blaZ* on the hybrid plasmid (rep5a and rep16) pJOR_*blaZ*. The two genes mediating the β-lactam resistance phenotype have been reported to be common determinants of antibiotic resistance in MRSA strains worldwide [46]. The limited data available in the literature indicate genotypic diversity within MRSA strains in different populations and environments in Jordan (Table 3). However, the current body of work may not fully reflect the entirety of MRSA’s landscape or even other ESKAP members, both in the community and within hospitals in Jordan [47]. It is important to maintain surveillance and conduct further investigation to obtain a comprehensive understanding of the present genotypes.

Isolate A29 contained various virulence factors, including genes *hlg(s), lukD/E, sak, scn, sea,* and *spl(s)*, that encode hemolysins, leukotoxins, staphylokinase, complement inhibitor, enterotoxin, and serine proteases, respectively. Most of these virulence factors are related to the infection process, except that the *scn* gene is considered an immune evasion marker, usually found in *S. aureus* isolated from humans. This gene was previously reported to help distinguish between strains of human origin and those of non-human origin [48]. Furthermore, a hybrid plasmid carrying *blaZ*, rep5a-rep16, was identified and blasted against the NCBI database. The *blaZ*-carrying plasmid was identical to that isolated from a Danish MRSA strain. Previous studies demonstrated a link between distinct *rep* sequences and resistance genotypes [49,50]. The PlasmidFinder detected that the plasmid pJOR_*blaZ* carried rep5a and rep16*,* the common plasmid replicon carrying *blaZ* gene [49]. 

## 5. Conclusions 

In conclusion, our study aimed to characterize the resistance profiles of 14 *S. aureus* isolates collected from various surfaces within Al-Karak governmental hospital, Jordan. Our utilization of whole-genome sequencing (WGS) on these isolates showed a novel spa type (t4407) that has never reported earlier in Jordan. High-quality data were obtained for only one isolate which is A29. The genomic data obtained for this isolate yielded valuable insights into the genetic traits for this MRSA strain. Notably, our analysis identified a novel MRSA type, spa type t4407 ST6-SCC*mec* type IVa, which had not been previously reported in Jordan. Furthermore, our bioinformatic analysis revealed a striking resemblance between the genome of isolate A29 and an MRSA isolate reported in Denmark in 2011. It is worth noting that the WGS of isolate A29 has enabled a comprehensive genetic analysis demonstrating the potential of this technology in uncovering the genetic characteristics of pathogens and clarifying their epidemiology. Nevertheless, it is still intriguing to consider what additional insights could be gained if high-quality data were obtained for the remaining isolates.

Therefore, future investigations involving comprehensive WGS of MRSA isolates in Jordan may offer a broader perspective on the genetic characteristics and transmission dynamics of this serious pathogen. Such endeavors hold promise for advancing our understanding of MRSA epidemiology and contributing to more effective strategies for its control and management.

## 6. Declaration

While preparing this work, the author(s) used ChatGPT to summarize texts. The tool was mainly used to improve the language style and readability not for generating lengthy text. After using this tool, the authors reviewed and edited the content as needed and took full responsibility for the publication’s content.

## Figures and Tables

**Table 1:** The genomic project properties for A29 MRSA isolated from Al-Karak Hospital, Jordan, 2019.

Property	
**Isolate name**	Alkarak2019-A29
Finishing quality	34
Libraries used	Nextera XT
Sequencing platforms	Illumina MiSeq
Fold coverage	20
Assembly method	SPAdes
GenBank ID	CP104989
GenBank date of release	23 February 2023
BIOPROJECT	PRJNA879252
BioSample	SAMN30801668
Locus tag	N4G46
Source material identifier	Inanimate object
Project relevance	Medical
**Genome properties**	
Genome size (bp)	2,789,696
DNA G + C content	33.5%
Number of contigs	56
N50	136,095
SEED subsystems (RAST annotation)	273
No. of coding sequences	2650
No. of RNAs	67

**Table 2:** Comparison of A29 genome to the nucleotide database NCBI, results of strain typing (SCC*mec* and spa), and identification of the plasmid replicon type.

**I. Whole-genome BLAST results**					
Hit no.	Query position in A29 genome	Closest hit (Acc. Code)	Identity (%)	Seq. length	Query Cover
1	Whole genome	CP047021.1	99.96	2791940	99%
**II. SCC*mec* Typing results**					
Hit no.	Query position in A29 genome	Closest hit (Acc. Code)	Identity (%)	Seq. length	Gene Name
1	2402…3892	AB063172	99.93	1491/1491	subtype-IVa (*2B*)
2	7844…9193	AB096217	100.00	1350/1350	*ccrA2*
3	9194…10,843	AB097677	99.94	1650/1650	*ccrB2*
4	14,516…15,502	AB033763	100.00	987/987	*dmecR1*
5	12,685…14,527	AM292304	100.00	1843/1843	*IS1272*
6	15,599…17,608	AB505628	100.00	2010/2010	*mecA*
**III. Acquired antimicrobial resistance genes**					
Hit no.	Query position in A29 genome	Closest hit (Acc. Code)	Identity (%)	Seq. length	Gene Name
1	15,616…17,626	BX571856	100	2010	*mecA*
2	2,694,466…2,695,311	AP004832	100	846	*blaZ*
**IV. Identification of the plasmid replicons**					
Hit	Query position in A29 genome	Closest hit (Acc. Code)	Identity (%)	Seq. length	*Replicon group*
*rep5A*	2,682,088…2,682,948	AP003139	100	861/861	Rep3
*rep16*	2,684,239…2,684,982	CP002115	100	744/744	Inc18
**V. *Spa* Typing results**					
Hit no.	Query position in A29 genome	Repeats			*spa* Type
1	2,734,797…2,735,002	11-10-21-17-34-22-25			t4407

**Table 3:** Comparative analysis of MRSA genotypic profiles reported in Jordan in the last decade, compared to the isolate currently reported (A29).

Reference No.	Reference	Sample Source	MLST	SCC*mec* Type (s)	Spa Type
[6]	Abdelmalek et al., 2022	University setting	ND	ND	ND
[9]	Al-Bakri et al., 2021	Diabetic foot ulcers	ND	III, IV, IVc, IVe	t044, t386, t267,t223,t018, t1339, t127, t311, t037, t605, t223, t9519
[10]	Al-Dmour et al., 2023	Hemodialysis patients, Al-Karak hospital	ND	ND	ND
[13]	Aqel et al., 2015	Healthcare workers	ND	IVa and Vc	t223
[15]	Darwish et al., 2022	Neonates, Healthcare professionals, Amman	ND	ND	t044, t012, t021, t223, t934, t253, t5075, t3534, t3767, t11023, t12492
[16]	Gharaibeh et al., 2023	Bovine mastitis	ND	ND	t17158
[17]	Jaradat et al., 2021	Public facilities, Northern Jordan	ND	ND	ND
[18]	Alzoubi et al., 2014	Children (6-11 years)	ND	IV	t223
[19]	Bazzoun et al., 2014	Hospital isolates	ND	ND	ND
[20]	Khalil et al., 2012	Children in Jordan	ST80	IV	ND
[43]	Harastani and Tokajian 2014	Clinical isolates, Amman	ST80, ST997	IV	t044, t5849, t5849, t5802, t6438
[44]	Al-Tamimi et al., 2021	Hospitalized patients	ND	ND	ND
This study	This study	Hospital objects, Al-Karak Hospital	ST6	IVa	t4407

ND: not determined.

## Data Availability

The data that support the findings of this study are available in NCBI at https://www.ncbi.nlm.nih.gov/nuccore/, reference number CP104989, accessed on 1 January 2024.

## Supplementary Materials

The following supporting information can be downloaded at: https://www.mdpi.com/article/10.3390/medicina60020295/s1, Figure S1. Annotation summary for MRSA strain Karak2019-A29 using RAST subsystem. Table S1. Virulence factors detected in A29 genome in the current study based on Virulence factor tool VirulenceFinder-2.0 Server (last accessed 14/1/2024). Table S2. SNP analysis of the A29 genome mapped to the antimicrobial resistance genes in CP047022.1 genome (Danish MRSA isolate).
