# Supplementary material for: Emergence and Genomic Characterization of a spa Type t4407 ST6-SCCmec Type IVa Methicillin-Resistant Staphylococcus aureus Strain Isolated from Al-Karak Hospital, Jordan
Source: Medicina (Kaunas). 2024 Feb 9;60(2):295. doi: 10.3390/medicina60020295 (PMC10890080; doi:10.3390/medicina60020295)
Supplement: Supplementary file 1 [file medicina-60-00295-s001.zip › medicina-2736203-supplementary.pdf]

medicina

Supplementary Information

Emergence and Genomic Characterization of a *spa* Type t4407 ST6-SCC*mec* Type IVa Methicillin-Resistant *Staphylococcus aureus* Strain Isolated from Al-Karak Hospital, Jordan

Yasser Gaber [1,2,*], Heba M. TumAllah [1], Nourhan H. AbdelAllah [2,3], Wael A. Al-Zereini [4], Mohammad A. Abu-Lubad [5], Amin A. Aqel [5], Walid F. Elkhatib [6,7], Richard V. Goering [8] and Ahmed M. Soliman [9]

[1] Department of Pharmaceutics and Pharmaceutical Technology, Faculty of Pharmacy, Mutah University,
Al-Karak 61710, Jordan; heba_tumallah@yahoo.com
[2] Department of Microbiology and Immunology, Faculty of Pharmacy, Beni-Suef University, Beni-Suef 62511, Egypt; nourhan.hassan@pharm.bsu.edu.eg
[3] Clinical Trials Department, Central Administrative of Biological, Innovative Products and Clinical Trials,
Egyptian Drug Authority, Giza 12654, Egypt
[4] Department of Biological Sciences, Faculty of Sciences, Mutah University, Al-Karak 61710, Jordan; wzereini@mutah.edu.jo
[5] Department of Microbiology and Pathology, Faculty of Medicine, Mutah University, Al-Karak 61710, Jordan; abu_lubbad@mutah.edu.jo (M.A.A.-L.); aminaq@mutah.edu.jo (A.A.A.)
[6] Microbiology and Immunology Department, Faculty of Pharmacy, Ain Shams University, African Union Organization St., Abbassia, Cairo 11566, Egypt; walid-elkhatib@gu.edu.eg
[7] Department of Microbiology & Immunology, Faculty of Pharmacy, Galala University, New Galala City, Suez 43713, Egypt
[8] Department of Medical Microbiology and Immunology, Creighton University School of Medicine,
Omaha, NE 68178, USA; richardgoering@creighton.edu
[9] Department of Microbiology and Immunology, Faculty of Pharmacy, Kafr-Elsheikh University,
Kafr El-Sheikh 33516, Egypt; ahmed_soliman@pharm.kfs.edu.eg

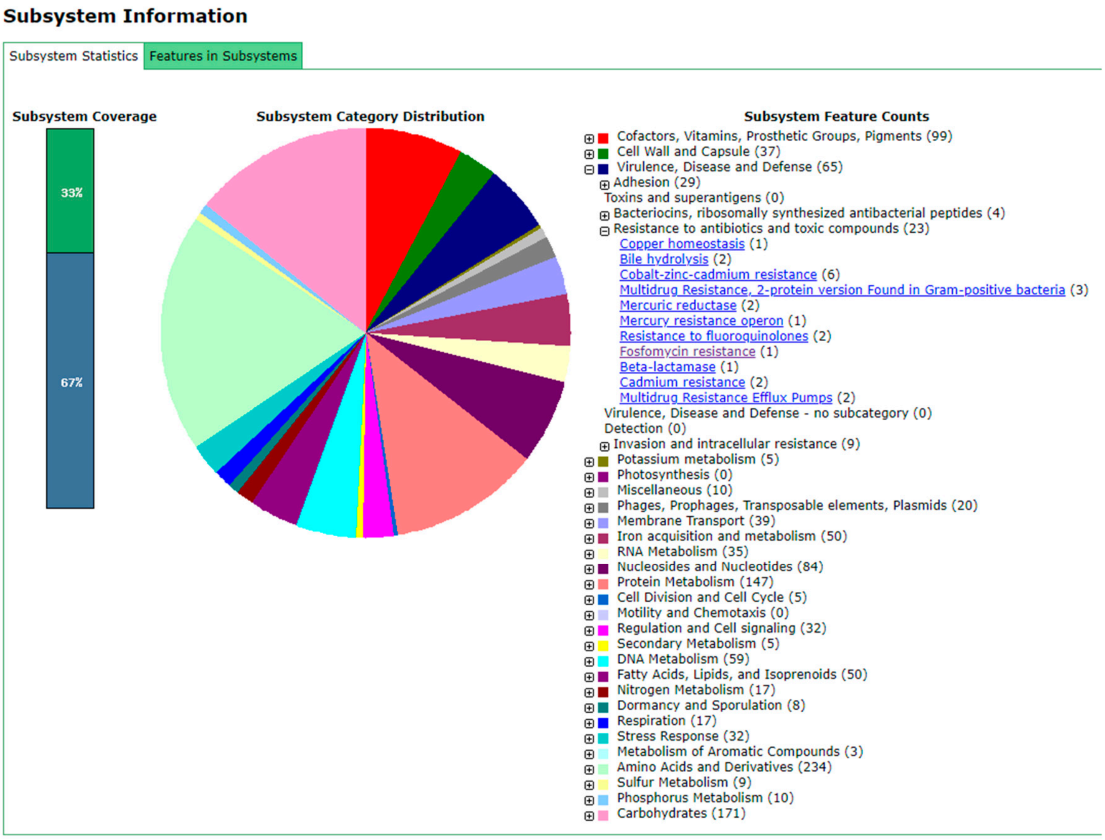

Figure S1. Annotation summary for MRSA strain Karak2019-A29 using RAST subsystem

Table S1 Virulence factors detected in A29 genome in the current study based on Virulence factor tool VirulenceFinder-2.0 Server (last accessed 14/1/2024)

Virulence factor	Identity	Query / Template length	Contig	Position in contig	Protein function	Accession number
**Host immunological genes for S. aureus.**						
sak	100	492 / 492	CP104989.1 Staphylococcus aureus strain Karak2019-A29 chromosome	2614676..2615167	staphylokinase	BA000017.4
scn	100	351 / 351	CP104989.1 Staphylococcus aureus strain Karak2019-A29 chromosome	2616664..2617014	staphylococcal complement inhibitor	LC032460.1
**Exoenzyme genes for S. aureus**						
**Virulence factor**	**Identity**	**Query / Template length**	**Contig**	**Position in contig**	**Protein function**	**Accession number**
aur	99.87	1530 / 1530	CP104989.1 Staphylococcus aureus strain Karak2019-A29 chromosome	145284..146813	aureolysin	AP014653.1
splA	99.86	708 / 708	CP104989.1 Staphylococcus aureus strain Karak2019-A29 chromosome	1122500..1123207	serine protease splA	AP014942.1
splB	99.72	723 / 723	CP104989.1 Staphylococcus aureus strain Karak2019-A29 chromosome	1123332..1124054	serine protease splB	AP014942.1
splE	100	717 / 717	CP104989.1 Staphylococcus aureus strain Karak2019-A29 chromosome	1125829..1126545	serine protease splE	AP009351.1
**Toxin genes for S. aureus**						
**Virulence factor**	**Identity**	**Query / Template length**	**Contig**	**Position in contig**	**Protein function**	**Accession number**
hlgA	100	930 / 930	CP104989.1 Staphylococcus aureus strain Karak2019-A29 chromosome	1345141..1346070	gamma-hemolysin chain II precursor	AP014942.1
hlgB	99.8	977 / 977	CP104989.1 Staphylococcus aureus strain Karak2019-A29 chromosome	1342648..1343624	gamma-hemolysin component B precursor	BA000033.2
hlgC	99.89	948 / 948	CP104989.1 Staphylococcus aureus strain Karak2019-A29 chromosome	1343626..1344573	gamma-hemolysin component C	AP014653.1
lukD	99.69	983 / 984	CP104989.1 Staphylococcus aureus strain Karak2019-A29 chromosome	1110128..1111110	leukocidin D component	AP014653.1
lukE	99.79	936 / 936	CP104989.1 Staphylococcus aureus strain Karak2019-A29 chromosome	1109191..1110126	leukocidin E component	AP014942.1

sea	100	783 / 783	CP104989.1 Staphylococcus aureus strain Karak2019-A29 chromosome	2612185..2612967	enterotoxin A	AP009324.1

medicina

MDPI

Table S2 SNP analysis of the A29 genome mapped to the antimicrobial resistance genes in CP047022.1 genome (Danish MRSA isolate)

Entry number	Start/Stope/SNP (Numbering according to CP047022.1 , the Danish Isolate genome	start/stop	Strand	Danish isolate	SNP deteced in A29 genome	Element symbol	Element name	Type	Scope	Subtype	Class	Subclass	Method	% Coverage of reference	% Identity to reference
1	3448	Start	+			cadD	cadmium resistance transporter CadD	STRESS	plus	METAL	CADMIUM	CADMIUM	BLASTP	100	99.51
2	4065	stop	+			cadD	cadmium resistance transporter CadD	STRESS	plus	METAL	CADMIUM	CADMIUM	BLASTP	100	99.51
3	8476	Start	-			blaR1	beta-lactam sensor/signal transducer BlaR1	AMR	core	AMR	BETA-LACTAM	BETA-LACTAM	BLASTP	100	95.56
4	10233	stop	-			blaR1	beta-lactam sensor/signal transducer BlaR1	AMR	core	AMR	BETA-LACTAM	BETA-LACTAM	BLASTP	100	95.56
5	10340	Start	+			blaZ	penicillin-hydrolyzing class A beta-lactamase BlaZ	AMR	core	AMR	BETA-LACTAM	BETA-LACTAM	EXACTP	100	100
6	11185	stop	+			blaZ	penicillin-hydrolyzing class A beta-lactamase BlaZ	AMR	core	AMR	BETA-LACTAM	BETA-LACTAM	EXACTP	100	100

Entry number	Start/Stope/SNP (Numbering according to CP047022.1 , the Danish Isolate genome	start/stop	Strand	Danish isolate	SNP deteced in A29 genome	Element symbol	Element name	Type	Scope	Subtype	Class	Subclass	Method	% Coverage of reference	% Identity to reference
7	39653	Start	-			mecA	PBP2a family beta-lactam-resistant peptidoglycan transpeptidase MecA	AMR	core	AMR	BETA-LACTAM	METHICILLIN	EXACTP	100	100
8	41662	stop	-			mecA	PBP2a family beta-lactam-resistant peptidoglycan transpeptidase MecA	AMR	core	AMR	BETA-LACTAM	METHICILLIN	EXACTP	100	100
9	41759	Start	+			mecR1	beta-lactam sensor/signal transducer MecR1	AMR	core	AMR	BETA-LACTAM	METHICILLIN	PARTIALP	55.56	100
10	42745	stop	+			mecR1	beta-lactam sensor/signal transducer MecR1	AMR	core	AMR	BETA-LACTAM	METHICILLIN	PARTIALP	55.56	100
11	61423	SNP in A29 genome		C	T										
12	87285	SNP in		C	T										

Entry number	Start/Stope/SNP (Numbering according to CP047022.1 , the Danish Isolate genome	start/stop	Strand	Danish isolate	SNP deteced in A29 genome	Element symbol	Element name	Type	Scope	Subtype	Class	Subclass	Method	% Coverage of reference	% Identity to reference
		A29 genome													
13	**117202**	SNP in A29 genome		G	T										
14	122824	Start	+			tet(38)	tetracycline efflux MFS transporter Tet(38)	AMR	core	AMR	TETRACYCLINE	TETRACYCLINE	EXACTP	100	100
15	124176	stop	+			tet(38)	tetracycline efflux MFS transporter Tet(38)	AMR	core	AMR	TETRACYCLINE	TETRACYCLINE	EXACTP	100	100
16	**129576**	SNP in A29 genome		G	A										
17	**195444**	SNP in A29 genome		C	T										

Entry number	Start/Stope/SNP (Numbering according to CP047022.1 , the Danish Isolate genome	start/stop	Strand	Danish isolate	SNP deteced in A29 genome	Element symbol	Element name	Type	Scope	Subtype	Class	Subclass	Method	% Coverage of reference	% Identity to reference
18	202792	SNP in A29 genome		T	G										
19	206133	SNP in A29 genome		A	C										
20	219993	SNP in A29 genome		G	T										
21	264964	SNP in A29 genome		A	G										
22	273908	SNP in A29 genome		C	T										
23	297162	SNP in		G	T										

Entry number	Start/Stope/SNP (Numbering according to CP047022.1 , the Danish Isolate genome	start/stop	Strand	Danish isolate	SNP deteced in A29 genome	Element symbol	Element name	Type	Scope	Subtype	Class	Subclass	Method	% Coverage of reference	% Identity to reference
		A29 genome													
24	351426	Start	+			mepA	multidrug efflux MATE transporter MepA	AMR	plus	AMR	TETRACYCLINE	TIGECYCLINE	BLASTP	100	99.33
25	352781	stop	+			mepA	multidrug efflux MATE transporter MepA	AMR	plus	AMR	TETRACYCLINE	TIGECYCLINE	BLASTP	100	99.33
26	385926	Start	+			selX	staphylococcal enterotoxin-like toxin X	VIRULENCE	plus	VIRULENCE			BLASTP	100	96.06
27	386537	stop	+			selX	staphylococcal enterotoxin-like toxin X	VIRULENCE	plus	VIRULENCE			BLASTP	100	96.06
28	**405800**	SNP in A29 genome		C	T										
29	**433226**	SNP in A29 genome		A	T										

Entry number	Start/Stope/SNP (Numbering according to CP047022.1 , the Danish Isolate genome	start/stop	Strand	Danish isolate	SNP deteced in A29 genome	Element symbol	Element name	Type	Scope	Subtype	Class	Subclass	Method	% Coverage of reference	% Identity to reference
30	459607	SNP in A29 genome		C	T										
31	466745	SNP in A29 genome		A	G										
32	471086	SNP in A29 genome		A	G										
33	478280	SNP in A29 genome		A	G										
34	496834	SNP in A29 genome		T	A										
35	557684	SNP in		A	G										

Entry number	Start/Stope/SNP (Numbering according to CP047022.1 , the Danish Isolate genome	start/stop	Strand	Danish isolate	SNP deteced in A29 genome	Element symbol	Element name	Type	Scope	Subtype	Class	Subclass	Method	% Coverage of reference	% Identity to reference
		A29 genome													
36	**594282**	SNP in A29 genome		G	A										
37	**596571**	SNP in A29 genome		T	C										
38	**623798**	SNP in A29 genome		G	A										
39	**651961**	SNP in A29 genome		C	T										
40	**788178**	SNP in A29		G	T										

Entry number	Start/Stope/SNP (Numbering according to CP047022.1 , the Danish Isolate genome	start/stop	Strand	Danish isolate	SNP deteced in A29 genome	Element symbol	Element name	Type	Scope	Subtype	Class	Subclass	Method	% Coverage of reference	% Identity to reference
		genome													
41	**824558**	SNP in A29 genome		A	T										
42	**827178**	SNP in A29 genome		A	G										
43	**847296**	SNP in A29 genome		G	A										
44	**848111**	SNP in A29 genome		A	G										
45	**862366**	SNP in A29 genome		G	A										

Entry number	Start/Stope/SNP (Numbering according to CP047022.1 , the Danish Isolate genome	start/stop	Strand	Danish isolate	SNP deteced in A29 genome	Element symbol	Element name	Type	Scope	Subtype	Class	Subclass	Method	% Coverage of reference	% Identity to reference
46	**1003956**	SNP in A29 genome		A	G										
47	**1008726**	SNP in A29 genome		T	C										
48	**1052634**	SNP in A29 genome		A	C										
49	**1055966**	SNP in A29 genome		C	T										
50	**1079677**	SNP in A29 genome		A	G										
51	**1094915**	SNP in		C	T										

Entry number	Start/Stope/SNP (Numbering according to CP047022.1 , the Danish Isolate genome	start/stop	Strand	Danish isolate	SNP deteced in A29 genome	Element symbol	Element name	Type	Scope	Subtype	Class	Subclass	Method	% Coverage of reference	% Identity to reference
		A29 genome													
52	1096368	SNP in A29 genome		C	A										
53	1100800	SNP in A29 genome		A	G										
54	1169946	SNP in A29 genome		T	C										
55	1265315	SNP in A29 genome		C	T										
56	1304832	SNP in A29		A	G										

Entry number	Start/Stope/SNP (Numbering according to CP047022.1 , the Danish Isolate genome	start/stop	Strand	Danish isolate	SNP deteced in A29 genome	Element symbol	Element name	Type	Scope	Subtype	Class	Subclass	Method	% Coverage of reference	% Identity to reference
		genome													
57	**1313113**	SNP in A29 genome		T	C										
58	**1337775**	SNP in A29 genome		G	A										
59	**1348357**	SNP in A29 genome		T	A										
60	**1358333**	SNP in A29 genome		A	G										
61	**1363484**	SNP in A29 genome		C	T										

Entry number	Start/Stope/SNP (Numbering according to CP047022.1 , the Danish Isolate genome	start/stop	Strand	Danish isolate	SNP deteced in A29 genome	Element symbol	Element name	Type	Scope	Subtype	Class	Subclass	Method	% Coverage of reference	% Identity to reference
62	1377084	SNP in A29 genome		C	T										
63	1410891	SNP in A29 genome		T	C										
64	1492314	SNP in A29 genome		T	C										
65	1494830	SNP in A29 genome		C	T										
66	1502240	SNP in A29 genome		A	G										
67	1510094	SNP in		C	T										

Entry number	Start/Stope/SNP (Numbering according to CP047022.1 , the Danish Isolate genome	start/stop	Strand	Danish isolate	SNP deteced in A29 genome	Element symbol	Element name	Type	Scope	Subtype	Class	Subclass	Method	% Coverage of reference	% Identity to reference
		A29 genome													
68	**1536586**	SNP in A29 genome		T	A										
69	**1555615**	SNP in A29 genome		T	C										
70	**1565266**	SNP in A29 genome		C	T										
71	**1585617**	SNP in A29 genome		T	C										
72	**1616832**	SNP in A29		A	T										

Entry number	Start/Stope/SNP (Numbering according to CP047022.1 , the Danish Isolate genome	start/stop	Strand	Danish isolate	SNP deteced in A29 genome	Element symbol	Element name	Type	Scope	Subtype	Class	Subclass	Method	% Coverage of reference	% Identity to reference
		genome													
73	**1632012**	SNP in A29 genome		A	G										
74	**1638378**	SNP in A29 genome		T	A										
75	**1642194**	SNP in A29 genome		T	C										
76	**1651495**	SNP in A29 genome		C	T										
77	**1746100**	SNP in A29 genome		T	C										

Entry number	Start/Stope/SNP (Numbering according to CP047022.1 , the Danish Isolate genome	start/stop	Strand	Danish isolate	SNP deteced in A29 genome	Element symbol	Element name	Type	Scope	Subtype	Class	Subclass	Method	% Coverage of reference	% Identity to reference
78	1746407	SNP in A29 genome		G	A										
79	1764608	SNP in A29 genome		A	G										
80	1778611	SNP in A29 genome		A	T										
81	1796127	Start	+			arsB	arsenite efflux transporter membrane subunit ArsB	STRESS	plus	METAL	ARSENIC	ARSENITE	HMM	100	81.82
82	1797419	stop	+			arsB	arsenite efflux transporter membrane subunit ArsB	STRESS	plus	METAL	ARSENIC	ARSENITE	HMM	100	81.82
83	1832210	Start	-			splE	serine protease SplE	VIRULENCE	plus	VIRULENCE			EXACTP	100	100
84	1832926	stop	-			splE	serine protease SplE	VIRULENCE	plus	VIRULENCE			EXACTP	100	100

Entry number	Start/Stope/SNP (Numbering according to CP047022.1 , the Danish isolate genome	start/stop	Strand	Danish isolate	SNP deteced in A29 genome	Element symbol	Element name	Type	Scope	Subtype	Class	Subclass	Method	% Coverage of reference	% Identity to reference
85	1834701	Start	-			splB	serine protease SplB	VIRULENCE	plus	VIRULENCE			BLASTP	100	99.58
86	1835423	stop	-			splB	serine protease SplB	VIRULENCE	plus	VIRULENCE			BLASTP	100	99.58
87	1835548	Start	-			splA	serine protease SplA	VIRULENCE	plus	VIRULENCE			EXACTP	100	100
88	1836255	stop	-			splA	serine protease SplA	VIRULENCE	plus	VIRULENCE			EXACTP	100	100
89	1848629	Start	-			lukE	bi-component leukocidin LukED subunit E	VIRULENCE	plus	VIRULENCE			BLASTP	100	99.68
90	1849564	stop	-			lukE	bi-component leukocidin LukED subunit E	VIRULENCE	plus	VIRULENCE			BLASTP	100	99.68
91	1869476	SNP in A29 genome		C	T										
92	1884962	SNP in A29 genome		T	C										

Entry number	Start/Stope/SNP (Numbering according to CP047022.1 , the Danish Isolate genome	start/stop	Strand	Danish isolate	SNP deteced in A29 genome	Element symbol	Element name	Type	Scope	Subtype	Class	Subclass	Method	% Coverage of reference	% Identity to reference
93	**1894879**	SNP in A29 genome		A	G										
94	**1899804**	SNP in A29 genome		T	A										
95	**1922068**	SNP in A29 genome		C	G										
96	**1965750**	SNP in A29 genome		G	A										
97	**2013482**	SNP in A29 genome		G	A										

Entry number	Start/Stope/SNP (Numbering according to CP047022.1 , the Danish Isolate genome	start/stop	Strand	Danish isolate	SNP deteced in A29 genome	Element symbol	Element name	Type	Scope	Subtype	Class	Subclass	Method	% Coverage of reference	% Identity to reference
98	2019010	Start	-			scn	complement inhibitor SCIN-A	VIRULENCE	plus	VIRULENCE			EXACTP	100	100
99	2019360	stop	-			scn	complement inhibitor SCIN-A	VIRULENCE	plus	VIRULENCE			EXACTP	100	100
100	2020858	Start	-			sak	staphylokinase	VIRULENCE	plus	VIRULENCE			EXACTP	100	100
101	2021349	stop	-			sak	staphylokinase	VIRULENCE	plus	VIRULENCE			EXACTP	100	100
102	2023058	Start	-			sea	staphylococcal enterotoxin type A	VIRULENCE	plus	VIRULENCE			EXACTP	100	100
103	2023831	stop	-			sea	staphylococcal enterotoxin type A	VIRULENCE	plus	VIRULENCE			EXACTP	100	100
104	**2028986**	SNP in A29 genome		G	A										
105	2077435	Start	-			hld	delta-hemolysin	VIRULENCE	plus	VIRULENCE			EXACTP	100	100
106	2077569	stop	-			hld	delta-hemolysin	VIRULENCE	plus	VIRULENCE			EXACTP	100	100
107	**2116396**	SNP in		T	C										

Entry number	Start/Stope/SNP (Numbering according to CP047022.1 , the Danish Isolate genome	start/stop	Strand	Danish isolate	SNP deteced in A29 genome	Element symbol	Element name	Type	Scope	Subtype	Class	Subclass	Method	% Coverage of reference	% Identity to reference
		A29 genome													
108	**2154783**	SNP in A29 genome		G	T										
109	**2156367**	SNP in A29 genome		C	T										
110	**2167776**	SNP in A29 genome		T	C										
111	**2194283**	SNP in A29 genome		A	G										
112	**2215118**	SNP in A29		A	G										

Entry number	Start/Stope/SNP (Numbering according to CP047022.1 , the Danish Isolate genome	start/stop	Strand	Danish isolate	SNP deteced in A29 genome	Element symbol	Element name	Type	Scope	Subtype	Class	Subclass	Method	% Coverage of reference	% Identity to reference
		genome													
113	2227623	Start	-			lmrS	multidrug efflux MFS transporter LmrS	STRESS	plus	BIOCIDE	MACROLIDE/PHENICOL	CHLORAMPHENICOL/ERYTHROMYCIN	BLASTP	100	99.38
114	2229065	stop	-			lmrS	multidrug efflux MFS transporter LmrS	STRESS	plus	BIOCIDE	MACROLIDE/PHENICOL	CHLORAMPHENICOL/ERYTHROMYCIN	BLASTP	100	99.38
115	**2244000**	SNP in A29 genome		A	G										
116	**2259380**	SNP in A29 genome		G	A										
117	**2265353**	SNP in A29 genome		T	G										
118	**2306928**	SNP in		A	G										

Entry number	Start/Stope/SNP (Numbering according to CP047022.1 , the Danish Isolate genome	start/stop	Strand	Danish isolate	SNP deteced in A29 genome	Element symbol	Element name	Type	Scope	Subtype	Class	Subclass	Method	% Coverage of reference	% Identity to reference
		A29 genome													
119	2309738	SNP in A29 genome		T	C										
120	2318631	SNP in A29 genome		T	C										
121	2323985	SNP in A29 genome		A	G										
122	2326107	SNP in A29 genome		T	C										
123	2328991	SNP in A29		G	A										

Entry number	Start/Stope/SNP (Numbering according to CP047022.1 , the Danish Isolate genome	start/stop	Strand	Danish isolate	SNP deteced in A29 genome	Element symbol	Element name	Type	Scope	Subtype	Class	Subclass	Method	% Coverage of reference	% Identity to reference
		genome													
124	2370143	SNP in A29 genome		T	C										
125	2371596	Start	+			fosB	FosB1/FosB3 family fosfomycin resistance bacillithiol transferase	AMR	core	AMR	FOSFOMYCIN	FOSFOMYCIN	PARTIALX	74.82	99.04
126	2371907	stop	+			fosB	FosB1/FosB3 family fosfomycin resistance bacillithiol transferase	AMR	core	AMR	FOSFOMYCIN	FOSFOMYCIN	PARTIALX	74.82	99.04
127	2387343	SNP in A29 genome		A	G										
128	2401737	SNP in A29		T	G										

Entry number	Start/Stope/SNP (Numbering according to CP047022.1 , the Danish Isolate genome	start/stop	Strand	Danish isolate	SNP deteced in A29 genome	Element symbol	Element name	Type	Scope	Subtype	Class	Subclass	Method	% Coverage of reference	% Identity to reference
		genome													
129	**2427776**	SNP in A29 genome		A	G										
130	**2440156**	SNP in A29 genome		A	G										
131	**2444821**	SNP in A29 genome		C	T										
132	**2459283**	SNP in A29 genome		A	G										
133	2462440	Start	+			hlgA	bi-component gamma-hemolysin HlgAB subunit A	VIRULENCE	plus	VIRULENCE			EXACTP	100	100

Entry number	Start/Stope/SNP (Numbering according to CP047022.1 , the Danish Isolate genome	start/stop	Strand	Danish isolate	SNP deteced in A29 genome	Element symbol	Element name	Type	Scope	Subtype	Class	Subclass	Method	% Coverage of reference	% Identity to reference
134	2463369	stop	+			hlgA	bi-component gamma-hemolysin HlgAB subunit A	VIRULENCE	plus	VIRULENCE			EXACTP	100	100
135	2463937	Start	+			hlgC	bi-component gamma-hemolysin HlgCB subunit C	VIRULENCE	plus	VIRULENCE			BLASTP	100	97.46
136	2464884	stop	+			hlgC	bi-component gamma-hemolysin HlgCB subunit C	VIRULENCE	plus	VIRULENCE			BLASTP	100	97.46
137	2464886	Start	+			hlgB	bi-component gamma-hemolysin HlgAB/HlgCB subunit B	VIRULENCE	plus	VIRULENCE			BLASTP	100	97.54
138	2465863	stop	+			hlgB	bi-component gamma-hemolysin HlgAB/HlgCB subunit B	VIRULENCE	plus	VIRULENCE			BLASTP	100	97.54
139	2467443	SNP in		C	T										

Entry number	Start/Stope/SNP (Numbering according to CP047022.1 , the Danish Isolate genome	start/stop	Strand	Danish isolate	SNP deteced in A29 genome	Element symbol	Element name	Type	Scope	Subtype	Class	Subclass	Method	% Coverage of reference	% Identity to reference
		A29 genome													
140	**2473513**	SNP in A29 genome		T	G										
141	**2509534**	SNP in A29 genome		T	C										
142	**2578503**	SNP in A29 genome		C	A										
143	**2597962**	SNP in A29 genome		T	C										
144	**2607734**	SNP in A29		C	T										

Entry number	Start/Stope/SNP (Numbering according to CP047022.1 , the Danish Isolate genome	start/stop	Strand	Danish isolate	SNP deteced in A29 genome	Element symbol	Element name	Type	Scope	Subtype	Class	Subclass	Method	% Coverage of reference	% Identity to reference
		genome													
145	**2618918**	SNP in A29 genome		C	T										
146	**2636282**	SNP in A29 genome		A	G										
147	**2649108**	SNP in A29 genome		G	T										
148	**2650162**	SNP in A29 genome		A	G										
149	2702407	Start	-			aur	zinc metalloprotease aureolysin	VIRULENCE	plus	VIRULENCE			BLASTP	100	99.41

Entry number	Start/Stope/SNP (Numbering according to CP047022.1 , the Danish Isolate genome	start/stop	Strand	Danish isolate	SNP deteced in A29 genome	Element symbol	Element name	Type	Scope	Subtype	Class	Subclass	Method	% Coverage of reference	% Identity to reference
150	2703936	stop	-			aur	zinc metalloproteinase aureolysin	VIRULENCE	plus	VIRULENCE			BLASTP	100	99.41
151	2703979	SNP in A29 genome		G	A										
152	2743489	SNP in A29 genome		G	A										
153	2747266	Start	+			icaC	polysaccharide intercellular adhesin biosynthesis/export protein IcaC	VIRULENCE	plus	VIRULENCE			EXACTP	100	100
154	2748318	stop	+			icaC	polysaccharide intercellular adhesin biosynthesis/export protein IcaC	VIRULENCE	plus	VIRULENCE			EXACTP	100	100
155	2748508	SNP in		A	C										

Entry number	Start/Stope/SNP (Numbering according to CP047022.1 , the Danish Isolate genome	start/stop	Strand	Danish isolate	SNP deteced in A29 genome	Element symbol	Element name	Type	Scope	Subtype	Class	Subclass	Method	% Coverage of reference	% Identity to reference
		A29 genome													
156	2758689	SNP in A29 genome		C	A										
157	2769798	Start	-			cna	collagen adhesin Cna	VIRULENCE	plus	VIRULENCE			BLASTP	100	98.22
158	2771818	SNP in A29 genome		A	G										
159	2773349	stop	-			cna	collagen adhesin Cna	VIRULENCE	plus	VIRULENCE			BLASTP	100	98.22
